# Antibiotic-Resistant ESKAPE Pathogens and COVID-19: The Pandemic beyond the Pandemic

**DOI:** 10.3390/v15091843

**Published:** 2023-08-30

**Authors:** Alessia Catalano, Domenico Iacopetta, Jessica Ceramella, Michele Pellegrino, Federica Giuzio, Maria Marra, Camillo Rosano, Carmela Saturnino, Maria Stefania Sinicropi, Stefano Aquaro

**Affiliations:** 1 Department of Pharmacy-Drug Sciences, University of Bari Aldo Moro, Via Orabona 4, 70126 Bari, Italy; 2 Department of Pharmacy, Health and Nutritional Sciences, University of Calabria, 87036 Arcavacata di Rende, Italy; domenico.iacopetta@unical.it (D.I.); jessica.ceramella@unical.it (J.C.); michele.pellegrino@unical.it (M.P.); mariamarra1997@gmail.com (M.M.); s.sinicropi@unical.it (M.S.S.); stefano.aquaro@unical.it (S.A.); 3 Department of Science, University of Basilicata, 85100 Potenza, Italy; federica.giuzio@unibas.it (F.G.); carmela.saturnino@unibas.it (C.S.); 4 Proteomics and Mass Spectrometry Unit, IRCCS Ospedale Policlinico San Martino, Largo Rosanna Benzi 10, 16132 Genova, Italy; camillo.rosano@hsanmartino.it

**Keywords:** SARS-CoV-2, COVID-19, antibacterials, post-COVID, ESKAPE, antibacterial resistance, DTR, MDR, XDR, PDR

## Abstract

Antibacterial resistance is a renewed public health plague in modern times, and the COVID-19 pandemic has rekindled this problem. Changes in antibiotic prescribing behavior, misinformation, financial hardship, environmental impact, and governance gaps have generally enhanced the misuse and improper access to antibiotics during the COVID-19 pandemic. These determinants, intersected with antibacterial resistance in the current pandemic, may amplify the potential for a future antibacterial resistance pandemic. The occurrence of infections with multidrug-resistant (MDR), extensively drug-resistant (XDR), difficult-to-treat drug-resistant (DTR), carbapenem-resistant (CR), and pan-drug-resistant (PDR) bacteria is still increasing. The aim of this review is to highlight the state of the art of antibacterial resistance worldwide, focusing on the most important pathogens, namely *Enterobacterales*, *Acinetobacter baumannii*, and *Klebsiella pneumoniae*, and their resistance to the most common antibiotics.

## 1. Introduction

The growing number of multidrug-resistant bacterial infections, which are undetected and undiagnosed, and the increasingly incurable infections threaten the health of people around the world [1]. While the COVID-19 pandemic has not yet left completely the scene, the antibacterial resistance is pressing. In 2015, a worldwide “Tripartite Alliance” effort of the three international bodies responsible for human health (World Health Organization, WHO), animal health (World Organization for Animal Health, WOAH), and food and agriculture (Food and Agriculture Organization of the United Nations, FAO), introduced a Global Action Plan (GAP) on antibacterial resistance [2]. Recently, the United Nations Environment Program (UNEP) has also been instituted, leading to a “Quadripartite Alliance”. Since 2015, countries around the world have developed and implemented their antimicrobial resistance (AMR) National Action Plans (NAPs). The “One Health Joint Plan of Action (2022–2026)” has been recently launched [3,4]. The general picture is now disastrous—not only AMR is still among us, but it also worsened during the pandemic, with infections very difficult to treat, which has increased the risk of spreading resistant strains, severe diseases, sepsis, and deaths. AMR and antibacterial resistance have been declared by the WHO as two of the top 10 public health threats worldwide [5]. Globally, the estimated number of deaths due to infections with multiple drug-resistant pathogens is approximately 700,000 annually [6]. In the article “Antimicrobial resistance: Tackling a crisis for the health and wealth of nations”, commissioned to Jim O’Neill by the British government and published in 2014, a catastrophic projection was given. It was estimated that by 2050, there will be approximately 10 million deaths per year caused by AMR, which is even higher than the sum of deaths from diabetes and cancer [7,8]. It is noteworthy that these alarming data refer to the pre-COVID era. The COVID-19 pandemic has strongly exacerbated many health problems [9], including AMR [10,11], leading to its designation as a “silent pandemic” [12,13], a “silent tsunami” or a “neglected pandemic” [14]. A growing body of evidence suggests that COVID-19 may impair the advances made in recent years [15,16,17,18,19,20,21]. However, in some cases, as in Japan, a substantial reduction in antimicrobial use was observed [22]. The prescription of antibiotics for COVID-19 hospitalized patients was imposed by the concern of bacterial superinfections or secondary bacterial pneumonia [23,24]. However, COVID-19 is a viral disease, and only a few patients might have bacterial co-infections; therefore, the use of antibiotics is not always necessary [25]. COVID-19 has increased the worldwide usage of antibiotics, personal protective equipment, and personal care products, such as soaps, handwashing liquids, and alcohol-based hand sanitizers, causing a knock-on effect in the existing global AMR problem [26,27,28,29,30]. Furthermore, the patients in intensive care, on prolonged mechanical ventilation, and with ventilator-associated pneumonia could have contributed to the colonization with nosocomial pathogens and led to an improved number of resistant isolates [31]. Nowadays, AMR has nearly leveled off in some high-income countries, but it continues to rise in low- and middle-income countries (LMICs), such as South Asia, South America, and Africa [32,33,34]. What will happen in the future is unknown. The definition of AMR as “a challenge awaiting the post-COVID-19 era” is somewhat concerning [35,36]. The awareness of dangers deriving from the use of too many antibiotics is useful, but in some cases, it is not enough to combat the possible resistance. For example, in a study conducted in Australia on children, it was demonstrated that parental knowledge of antibacterial resistance does not generally translate into a responsible use of antibiotics for their children [37]. The inappropriate consumption and application of antibiotics have driven the rapid emergence of diverse resistant Gram-negative bacteria, including PDR, DTR, XDR, MDR, and CR Gram-negative bacteria (Table 1) [38,39,40].

Much of the morbidity and mortality related to antibacterial resistance is due to the nosocomial emergence of a group of pathogens designated with the acronym “ESKAPE” by the Hospital and Infectious Diseases Society of America (IDSA) [41]. ESKAPE pathogens are represented by both Gram-positive and Gram-negative bacteria, namely, *Enterococcus faecium*, *Staphylococcus aureus*, *K. pneumoniae*, *A. baumannii*, *Pseudomonas aeruginosa*, and *Enterobacter* spp. [42,43,44]. In the COVID-19 and post-COVID scenarios, they are intersected [45,46]—the co-infections with Gram-negative ESKAPE bacteria are more frequent in patients with severe COVID-19 symptoms than in patients with milder symptoms [47]. Resistance to antibiotics, such as colistin, tigecycline, and carbapenems, earns ESKAPE pathogens the classification as bacteria needing particular attention by the WHO and as an urgent threat to public health by the Centers for Disease Control and Prevention (CDC) [48]. Finally, the effects of wars and climate change may be the accelerator factors as well [49,50]. In this review, we summarize the most common strains of ESKAPE bacteria resistant to antibiotics and employed therapies. We also highlight some recent studies in relation to the effect registered on antibacterial resistance by the occurrence of the pandemic worldwide.

## 2. MDR, XDR, PDR and DTR Bacteria

Different types of bacteria resistant to antibiotics have been described worldwide. The most common Gram-negative resistant bacteria are listed in Table 1. MDR bacteria are those bacteria that gained resistance to three or even more agents; XDR pathogens are resistant to all drugs, except to colistin and tegacyclin, whereas PDR bacteria are resistant to all antibiotics (even including colistin and tegacyclin). Finally, difficult-to-treat resistance (DTR) encompasses resistance to all first-line agents, including all β-lactams (carbapenems and β-lactamase inhibitor combinations) and fluoroquinolones [51]. Extended-spectrum beta-lactamases (ESBLs) were first described in 1983 and are able to hydrolyze penicillins, third-generation cephalosporins, and also the monobactam aztreonam [52]. ESBL-producing *Enterobacterales* (EPE) and carbapenemase-producing *Enterobacterales* (CPE) are the most commonly described and often display multidrug-resistant phenotypes [53,54]. The diverse types of resistance, PDR, DTR, XDR, MDR, CR, and ESBL are related to bacteria; resistant Gram-negative bacteria are listed in Table 2 and indicated with acronyms based on the name of the bacterium. They include *P. aeruginosa*, *A. baumannii*, *K. pneumoniae*, *Enterobacter* spp., and *E. coli*. Treatment for ESBL-producing *Enterobacterales* (ESBL-E), carbapenem-resistant *Enterobacterales* (CRE), and *P. aeruginosa* (CR-Pa) with DTR-P bacteria have been recently reviewed [55]. Both the CDC and WHO have emphasized that CR Gram-negative pathogens, including *Enterobacterales*, *P. aeruginosa*, and *A. baumannii*, were the major healthcare threats worldwide [48,56]. In 2017, the WHO included CR *K. pneumoniae* (CR-Kp) and *A. baumannii* (CR-Ab) as critical priorities for research and drug development [57]. The latter are opportunistic pathogens very often mentioned in the literature because they are frequently co-isolated from polymicrobial infections [58]. Infections caused by MDR Gram-negative bacteria are generally associated with a poor prognosis and an increased fatality rate of over 40%, especially in the presence of septic shock [59].

## 3. *Enterobacter* spp., *A. baumanni*, and *K. pneumoniae*

*Enterobacter* spp., *A. baumanni*, and *K. pneumoniae* are among the most prevalent strains of serious concern in nosocomial pneumonia, complicating the management of ventilated intensive care unit (ICU) patients [60]. Their spread in medical devices, patients, and medical personnel of the ICUs for COVID-19 patients has led to infections and sometimes deaths of many patients [13]. 

### 3.1. The Genus Enterobacter

The genus *Enterobacter* belongs to the *Enterobacteriaceae* family, which is the largest group of the order Enterobacterales (often erroneously reported as Enterobacteriales) [61]. Particularly, *E. aerogenes*, *E. cloacae*, and *E. hormaechei* represent the most frequently isolated species described in clinical infections, especially in immunocompromised and hospitalized ICU patients, due to their adaptation to drugs and their behavior as opportunistic pathogens. These species have an intrinsic resistance to ampicillin, amoxicillin, first-generation cephalosporins, and cefoxitin due to the expression of a constitutive AmpC β-lactamase. Furthermore, they produce ESBLs, which makes their treatment more difficult [62,63].

### 3.2. A. baumannii

*A. baumannii* is the most representative bacterium of the genus *Acinetobacter*, which has received great attention since the emergence of COVID-19 [64]. The most important agent belonging to this genus is *A. baumannii*, an aerobic Gram-negative opportunistic bacterium that emerged as a highly problematic pathogen for many healthcare institutions. This is a ubiquitous, Gram-negative, non-flagellated coccobacillus commonly isolated from the environment. The name *baumannii* was due to the researcher Baumann who published a comprehensive survey about this genus in 1968 [65]. Its clinical significance, mainly in the last 15 years, has been related to its remarkable ability to acquire resistance determinants, leading to its consideration as one of the organisms threatening the current antibiotic era [66]. Unfortunately, numerous *A. baumannii* strains resistant to all known antibiotics have now been reported. Moreover, this emerging resistance profile is in synergism with the uncanny ability of *A. baumannii* to survive for prolonged periods in a hospital environment [67]. It privileges the seriously ill within ICUs and results in a range of infectious syndromes in military personnel injured in the Iraq and Afghanistan conflicts [68] and more recently in the war in Ukraine [69]. The capability of *A. baumannii* to form biofilms and resist desiccation, antiseptics, and disinfectants is even more alarming, as these characteristics enable *A. baumannii* to develop within hospital settings. Moreover, in the study by Whiteway et al. (2022) [70], a high heterogeneity among isolates was observed at the genetic level due to a plastic genome, thus adding complexity to the study of *A. baumannii* as an entity. The current status of *Acinetobacter* infections has been recently reported by Nocera and De Martino (2023) [71]. Resistance to diverse antibiotics has been shown by *A. baumanni*. MDR bacteria in patients with war-related injuries, including CR-Ab, are a well-described issue of concern [72]. CR-Ab is an opportunistic pathogen primarily associated with hospital-acquired infections and seems to be the main pathogen involved, with a higher incidence compared with the pre-COVID period and a higher risk of death than other MDR organisms [73,74,75]. CR-Ab easily contaminates the hospital environment and the hands of healthcare workers as it may survive for prolonged periods on dry surfaces and can diffuse by asymptomatic colonization. For these reasons, it is often difficult to control CR-Ab outbreaks in acute care hospitals [76]. CR-Ab is resistant to common disinfectants [77] and is the primary cause of morbidity and mortality in several countries [78]. In hospitals with both ICU and non-ICU settings, stringent adherence to infection control practices is crucial to discontinue the transmission of CR-Ab. In the work by Zhang et al. (2020) [79], carried out in China, approximately 55.6% of COVID-19 patients were co-infected with CR-Ab in the ICU. Despite the fact that MDR-Ab and XDR-Ab represent a critical cause of healthcare-associated infections worldwide, the existing treatment options are very limited, and colistin-based therapies are last-line treatments for these types of infection, even though colistin-resistant (COL^R^) *Ab* have rarely been isolated yet. In bacteria, small noncoding RNAs (sRNAs) have been involved in regulatory pathways of diverse biological functions; however, no knowledge exists about the sRNA’s role in the biological adaptation in COL^R^ *Ab* [80].

### 3.3. K. pneumoniae 

*K. pneumoniae* is a Gram-negative bacterium belonging to the Enterobacteriaceae family, encapsulated and nonmotile, which resides in the environment, including soil and surface waters, and on medical devices [81,82]. *K. pneumoniae* was first isolated in the late 19th century and was initially known as Friedlander’s bacterium [83,84]. It is typically a gut commensal that can reside in the gastrointestinal tract and oropharynx as a physiological component of the intestinal flora and on the skin. In these sites, the effects of its colonization appear benign. However, *K. pneumoniae* strains can enter other tissues and cause severe infections in humans [85], such as urinary tract infections, cystitis, pneumonia, surgical wound infections, endocarditis, and septicemia, especially in immunocompromised patients [86]. It is also an important cause of serious community-onset infections, such as necrotizing pneumonia, pyogenic liver abscesses, and endogenous endophthalmitis [87]. The pathogenicity of *K. pneumoniae* is related to the envelope, lipopolysaccharides, and cell wall receptors, which define the process of binding to host cells and give protection against response from the human immune system [88]. These bacteria frequently demonstrate resistance to antibiotics of the carbapenem group in the course of severe infections caused by Gram-negative bacilli [89,90]. Recently, *K. pneumoniae* has been responsible for a common bacterial co-infection in hospitalized patients with COVID-19 [91,92,93]. The importance of *K. pneumoniae* as a leading causal agent of CRE urinary tract infections (UTIs), both before and during the COVID-19 pandemic, has been recently validated [94]. It may exist in two main pathotypes: classical *K. pneumoniae* (cKp) and hypervirulent *K. pneumoniae* (hvKp). cKP is a well-known frequent Gram-negative nosocomial pathogen that may cause pneumonia, urinary tract infections, meningitis, and sepsis in immunocompromised patients [95]. hvKp was originally recognized as the pathogen responsible for severe community-acquired infections among relatively healthy individuals, termed “invasive syndrome” [96,97,98]. hvKp isolates carry virulence plasmids harboring cardinal virulence genes, with frequencies superior to the classical *K. pneumoniae*, and they cause diffuse infections concerning the liver, lungs, central nervous system, and eyes [99]. hvKp colonizes the gastrointestinal tract, contributing to its spread in the community and healthcare settings [100]. It was first recognized as a cause of pyogenic liver abscesses in East Asia [101], and in recent years, sporadic cases have been increasingly reported worldwide [102], including India [103], China [104], Europe [105,106], Australia [107], and the United States [108,109]. Moreover, recent studies have suggested an increased rate of MDR in hvKp species, previously identified predominantly in nosocomial cKp infections [110,111]. Thus, the CDC and the WHO declared such emerging MDR and hvKp infections as an urgent public health threat [112,113,114]. These infections may seriously complicate the course of COVID-19, especially in hvKp-endemic areas. Superimposed hvKp infection with COVID-19 has been reported in Japan [92].

## 4. Therapies for Antibiotic-Resistant Bacteria

Carbapenems (doripenem, ertapenem, imipenem, meropenem, Table 3) were considered the most appropriate and potent agents to treat Gram-negative infections [115]. However, the widespread of CR Gram-negative bacteria rapidly evolved worldwide [116]. In the global priority list of antibiotic-resistant bacteria published by the WHO in 2017, three out of four pathogens with critical priority for new antibiotic development are CR pathogens, including CRE, CR-Pa, and CR-Ab [114]. Some noncarbapenem agents, including colistin (or polymyxin E) [117] and tigecycline [118], and some Gram-negative bacteria acquired resistance to these drugs [119,120]. Nowadays there are new therapies adopted against CR and colistin-resistant Gram-negative bacteria, including synergistic antimicrobials [121], such as clofoctol [122], capric acid [123], flufenamic acid [124] and polymyxyn [38]. 

Diverse new agents active against certain CR pathogens, including ceftazidime-avibactam [125], ceftolozane-tazobactam [126,127], piperacillin-tazobactam [128], meropenem-vaborbactam [129], imipenem-relebactam [130], imipenem-cilastatin-relebactam [131] are used in clinics. Specifically, plazomicin [132], eravacycline [133], and cefiderocol [134] have been approved for clinical use or are reaching late-stage clinical development [135]. However, some Gram-negative MDR bacilli acquired resistance as well [136]. For the treatment of *A. baumanni* resistant infections, recent studies reported the use of β-lactam-β-lactamase inhibitor combination (sulbactam-durlobactam), specifically for the treatment of CR-Ab [137]. Recently, alternative strategies for the treatment of MDR infections in human clinical settings have been reviewed [138]. They include combination therapies and techniques targeting the enzymes or proteins responsible for antimicrobial-resistant bacteria, bacteriophages, and their lytic enzymes [139]. Specifically, CRISPR-Cas (clustered regularly interspersed short palindromic repeats—CRISPR-associated protein) systems are genomic engineering tools targeting quantitatively, specifically, and selectively bacterial genomes to lower or eliminate the resistance, discriminating between pathogenic and commensal bacteria [140]. Moreover, besides antibiotics, nonantibiotic treatment strategies are being investigated for the treatment of bacterial resistance. Recently, new research on *A. baumannii* vaccines, specifically whole-cell vaccines, including inactivated and live attenuated bacterial ghost and DNA ones, has been reported [141]. Infections caused by extensively drug-resistant *A. baumannii* (XDR-Ab) and plasmid/chromosomal-mediated (Col-R-*Ab* COL^R^-*Ab*) are the main challenge and need a well-planned control program and proper treatment [142,143].

## 5. Co-Infections and Secondary Bacterial Infections in the COVID-19 Outbreak

Co-infections and secondary bacterial infections are known complications of viral respiratory infections and are dramatically associated with poorer outcomes in COVID-19 patients, despite antibiotic treatments [144]. During the COVID-19 pandemic, several immunocompromised patients were hospitalized and diagnosed with co-infections and secondary infections [145]. The incidence, prevalence, and characteristics of bacterial co-infection were not easily understood at the beginning, representing an important knowledge gap [146]. As reported by Kariyawasam et al. (2022) [147] in a systematic review and meta-analysis relative to the first 18 months of the pandemic (November 2019–June 2021), the prevalence of AMR in COVID-19 patients depends on variation between hospitals and geographic settings [148]. Particularly, bacterial and fungal co-infections and superinfections had a critical role in the outcome of the COVID-19 patients admitted to the ICUs [149]. The risk for secondary infection was found substantially greater than the risk for co-infection [150]. Bacterial co-infection and secondary infection in patients with COVID-19 were found to be 3.5% and 14.3%, respectively. In general, bacterial infection was 6.9%, varying slightly in the patient population, ranging from 5.9% in hospitalized patients to 8.1% in critically ill patients [151]. In other studies, similar results were reported, with only 7% of hospitalized patients showing bacterial co-infections with a high degree of heterogeneity, increasing to 14% for ICU patients [152,153]. Commonly identified co-pathogens of SARS-CoV-2 were *Streptococcus pneumoniae*, *S. aureus*, *K. pneumoniae*, *Haemophilus influenzae*, *Mycoplasma pneumoniae*, *A. baumannii*, *Legionella pneumophila*, and *Clamydia pneumoniae*, followed by coronavirus, rhinovirus/enterovirus, parainfluenza, metapneumovirus, influenza B virus, and human immunodeficiency virus [154]. Among bacteria, once again, *K. pneumoniae* and *A. baumannii* were recognized as the most common pathogens [71,155,156,157] and often co-isolated from polymicrobial infections. The co-infections can be more severe and obstinate to therapy than infections caused by either species alone. A recent study by Semenec et al. [58] suggested the existence of distinct syntrophic interactions between *A. baumannii* and *K. pneumoniae*. The authors characterized the genomes of both strains co-isolated from a single human lung infection and investigated several aspects of their interactions through transcriptomic, phenomic, and phenotypic assays. It was demonstrated that *K. pneumoniae* was able to cross-feed *A. baumannii* by-products of sugar fermentation, while *A. baumannii* was able to cross-protect *K. pneumoniae* against the cephalosporin and cefotaxime. The characteristics of the *A. baumannii* SARS-CoV-2 co-infection were not clearly defined. Genomic analyses of several MDR *A. baumannii* clinical strains were studied; for instance, the *A. baumannii* AMA_NO strain isolated in 2021 from a patient with COVID-19 was compared with the AMA166 isolated from a mini-BAL on a patient with pneumonia in 2016 [158]. A recent study by Saleh Ahmed et al. evidenced a high incidence rate of genetically related MDR *A. baumannii*, especially in active age group male patients. The drug-resistant *A. baumannii* could be tigecycline-sensitive; however, antibiotic susceptibility testing in COVID-19 patients is strongly recommended in order to preclude the wrong prescription of antibiotics and resistance phenomena [159]. Boorgula et al. [160] recently suggested that it is crucial to endorse antimicrobial stewardship programs and timely re-examine the empirical antibiotic policy to contain secondary infections in COVID-19 patients.

## 6. State of the Art in the World

The global impact of COVID-19 on bacterial resistance worldwide was widely described [161], and the following paragraph summarizes some significative studies regarding antibacterial resistance related to COVID-19 around the globe [162].

### 6.1. Asia

The WHO Southeast Asia Region runs the highest risk for AMR emergence among all WHO regions in Asia, given the inadequate funding or availability of funds for a short period of time; thus, it was termed a “global hub for AMR emergence” [163] and may potentially become a two-edged sword after the COVID-19 pandemic era [164,165]. Vijay et al. (2021) [166] reported a retrospective study of secondary bacterial infections in 17,534 patients admitted to ICUs and wards of ten hospitals of the Indian Council of Medical Research between June and August 2020. Among the patients who died of secondary infections, 72% had Gram-negative infections, 10.8% had Gram-positive infections, and 8% had mixed infections with Gram-positive and Gram-negative pathogens. Among Gram-negative bacteria, *K. pneumoniae* was the predominant pathogen, followed by *A. baumannii* (29% and 21%, respectively). High levels of resistance to carbapenems were seen in *A. baumannii*, followed by *K. pneumoniae* (CR-Ab, 92.6% and CR-Kp, 72.8%, respectively). Saini et al. (2021) [167] reported a study on blood and urine samples obtained from COVID-19 patients admitted to the ICU and investigated in the Department of Microbiology of a tertiary care hospital in Delhi, India. The most common blood isolates were coagulase negative *Staphylococcus* and *S. aureus*, while among the urinary isolates, the most common pathogens were *E. coli* and *S. aureus*. Regarding Gram-negative bacteria, *A. baumannii* emerged as the predominant bacteria isolated during COVID-19 (followed by *E. coli* and *K. pneumoniae*) compared with the pre-COVID-19 period, in which *K. pnemoniae*, followed by *Acinetobacter* spp., *Escherichia coli*, and *P. aeruginosa*, were the common blood isolates. During the pandemic, *A. baumanni* showed a reduced susceptibility to gentamicin, amikacin, and ciprofloxacin and an alarmingly lower susceptibility to cotrimoxazole and piperacillin-tazobactam. Ahmed et al. (2022) [168] recently described a study carried out in a tertiary care hospital in Lahore, Pakistan, among 1165 hospitalized COVID-19 patients, 423 of whom were found to be positive for different bacterial infections. Most of the isolated pathogens were Gram-negative, followed by Gram-positive bacteria (*n* = 366 and 57, respectively). Among them, *S. aureus* showed high resistance against tetracycline (61.7%), *S. pyogenes* was 100% resistant to penicillin, *E. coli* showed high resistance against ampicillin-clavulanic acid (88.72%), *P. aeruginosa* against ciprofloxacin (75.40%), whereas *Klebsiella pneumoniae* was 100% resistant to ampicillin. *A. baumannii* was 100% resistant to most of tested antibiotics. These data clearly indicate an alarming rise in antibacterial resistance during the pandemic on this continent.

### 6.2. Europe

In the WHO European Region, antibacterial resistance represents a major public health concern: the EU/EEA (European Union/European Economic Area) reported that more than 670,000 infections per year are due to bacterial resistance. Each year, resistance phenomena are responsible for about 33 000 deaths and cost about EUR 1.1 billion to the healthcare systems of EU/EEA countries [169]. A comparison between data before and after COVID-19 has been recently reported in a review by Khaznadar et al. (2023) [170].

The Organization for Economic Cooperation and Development (OECD) predicted that in 2030, resistance to second-line antibiotics will be 72% higher compared with 2005. The resistance phenomena in Europe are variable, with a gradient north-to-south and west-to-east, with higher percentages in the southern and eastern parts of Europe. Moreover, resistance to third-generation cephalosporins and carbapenems in *K. pneumoniae* and CR-Ab and CR-Pa in several countries in the European Region is of concern [171].

Gysin et al. (2021) [172] described a study relative to the period March–May 2020 on 70 Gram-negative bacterial strains, isolated from the lower respiratory tract of ventilated COVID-19 patients in Zurich, Switzerland. *P. aeruginosa* and *Enterobacterales* were the two mostly represented etiologic groups (46% and 36%, respectively). *P. aeruginosa* isolates were resistant to piperacillin-tazobactam (65.6%), cefepime (56.3%), ceftazidime (46.9%), and meropenem (50.0%). *Enterobacterales* isolates showed lower levels of resistance to piperacillin/tazobactam, ceftriaxone, and ceftazidime (about 30%). Cogliati Dezza et al. (2022) [173] conducted an observational, retrospective, single-center study, including patients admitted to the same ICU of an academic tertiary hospital in Rome, Italy, to evaluate the impact of COVID-19 on MDR Gram-negative bacteria bloodstream infections (BSIs). Non-COVID-19 patients had a higher incidence of MDR Gram-negative BSIs and were more likely to present *K. pneumoniae* BSIs, while the COVID-19 group showed more *A. baumannii* BSIs, with higher incidence per pathogen. Falcone et al. (2022) [174] reported a prospective, observational study including consecutive COVID-19 patients with hvKp infections admitted to the University Hospital of Pisa (Italy), from November 2020 to March 2021. hvKp was isolated from 36 COVID-19 patients: 80.6% had infections and 19.4% had colonization. The hvKp isolates displayed an ESBL phenotype, showing resistance to piperacillin/tazobactam and ceftolozane/tazobactam and susceptibility only to meropenem and ceftazidime/avibactam. Most patients were treated with meropenem alone or in association with fosfomycin. Thirty-day mortality was high, specifically 48.3%.

### 6.3. Africa 

COVID-19 spread rapidly and extensively to Africa [175,176]. In Africa, as in other LMICs, bacterial resistance is strongly correlated with poor governance and transparency more than with factors such as antibiotic use. Many African countries have National Action Plans (NAPs), but it is not clear whether information is publicly available on their implementation, monitoring, and financing [177].

Mutua et al. (2021) [178] reported a descriptive cross-sectional study design on severely ill COVID-19 patients at Kenyatta National Hospital, Kenya, between October and December 2021. A high incidence of bacterial infections was found in hospitalized COVID-19 patients during the peak of the pandemic. Interestingly, men were more susceptible than women. Patients of advanced age, not vaccinated, admitted to the critical care unit, and patients with prolonged length of hospital stay showed poor hospitalization outcomes. The majority of bacteria isolates (64.3%) were MDR, mainly Gram-negative bacteria (69.6%). The predominant MDR phenotypes were found in *Enterococcus cloacae* (42.9%), *K. pneumoniae* (25%), and *E. coli* (40%) and mostly involved cefotaxime, ceftriaxone, gentamicin, ciprofloxacin, aztreonam and trimethoprim/sulfamethoxazole. Adebisi et al. (2021) [179] reported a review on the use of antibiotics in COVID-19 management in 10 African countries, namely, Ghana, Kenya, Uganda, Nigeria, South Africa, Zimbabwe, Botswana, Liberia, Ethiopia, and Rwanda. In the study, it was evidenced that several antibiotics, including azithromycin, doxycycline, clarithromycin, ceftriaxone, erythromycin, amoxicillin, amoxicillin-clavulanic acid, ampicillin, gentamicin, benzylpenicillin, piperacillin/tazobactam, ciprofloxacin, ceftazidime, cefepime, vancomycin, meropenem, and cefuroxime among others, were recommended for use in the management of COVID-19 opportunistic bacterial infections. The authors underlined that this was worrisome because COVID-19 is a viral disease, and only a few patients had real bacterial co-infection, highlighting the cautious and judicious use of antibiotics. 

### 6.4. Australia

Australia is a nation with relatively low levels of antibacterial resistance due to its geographical isolation and community and agricultural stewardship [180]. However, the overuse or inappropriate prescribing of antibiotics in health (humans and animals) and agricultural sectors is a concern that has been enhanced over the years [181]. 

A study of the five resistant hospital-associated infections in Australia reported that in 2020, there were 1031 antimicrobial-resistant-associated deaths, more than the number of deaths (953) caused by influenza in 2019 [182]. The impact that the pandemic had on resistance phenomena in Australia is not indicative since the worst effects of the COVID-19 pandemic were avoided with an initial maximum suppression strategy [183]. Some authors report that constructive journalism, telling complex and scary stories in accessible ways, was crucial for listeners and may have promoted behavioral change around health issues [184]. 

A recent study compared patients admitted before the SARS-CoV-2 pandemic (1 July 2019–29 February 2020) and during the SARS-CoV-2 pandemic (1 March 2020–30 October 2021), 5.1% of which were SARS-CoV-2 positive. Overall resistance rates were not significantly increased from pre-pandemic levels; however, higher rates were observed in SARS-CoV-2–positive hospital-onset infections [185].

### 6.5. America

According to the annual Tripartite AMR Country Self-Assessment Survey 2020–21 in the USA, 94% of countries (151 out of 161) indicated that the pandemic impacted their national response to tackling AMR [186]. Recently, a Landscape Analysis Tool (LAT) was developed, supporting seven South American countries (Argentina, Brazil, Chile, Colombia, Paraguay, Peru, and Uruguay), in order to improve One Health activities and strengthen National Action Plans to curb AMR [187].

*N. gonorrhoeae*, the cause of the sexually transmitted infection gonorrhea, is a crucial public health threat in the United States [188]. However, in an analysis of gonorrhea trend during the pandemic compared with the pre-COVID era in one U.S. urban area, no increase in the proportion of reported diagnoses was observed despite the decreased screening [189]. In a study carried out in Mexico in 46 medical centers, an increase in methicillin-resistant *S. aureus*, carbapenem-resistant *K. pneumoniae*, and antibiotic-resistant *A. baumannii* and *P. aeruginosa* were observed during the COVID-19 pandemic [190].

However, data are often discordant, as recently reported by Gandra et al. (2023) [191]. The impact of the COVID-19 pandemic on resistance phenomena was studied in a community hospital in India and two community hospitals (Hospitals A and B) in St. Louis, MO, USA. In the Indian hospital, the prevalence of CR-Kp and CR-Ec was significantly higher during the pandemic period. In hospital A, the prevalence of methicillin-resistant *S. aureus* was higher during the COVID-19 pandemic, whereas in hospital B, the was no significant rise in MDR Gram-negative bacteria.

In several countries in Latin America and the Caribbean, the clinical emergence of CPEs, previously not characterized, was reported during the first wave of COVID-19 (during 2020–2021) [192]. A case-control study was carried out in Brazil from March 2020 to December 2021 to evaluate factors associated with the acquisition of MDR Gram-negative bacteria in patients with and without COVID-19. The study showed that mortality was significantly higher in COVID-19 patients infected with MDR Gram-negative bacteria compared with control groups with one of the two diseases, particularly among critically ill patients [193].

## 7. Conclusions

The emergence and global expansion of antibacterial resistance is an increasing healthcare threat worldwide. The COVID-19 pandemic gave the opportunity to rethink and strengthen the fight against the spread of resistance phenomena, promoting a culture of fair risk and the fight against transmissible diseases, but this objective has not yet been minimally reached. The ESKAPE pathogens, a ‘critical’ category of bacteria with rapid antibacterial resistance development, such as *E. faecium*, *S. aureus*, *K. pneumoniae*, *A. baumannii*, *P. aeruginosa*, and *Enterobacter* species, have been investigated during and after the pandemic. The large number of published studies regarding this issue are not in agreement with regard to a direct link between COVID-19 and more severe bacterial resistance. Indeed, it is not clear whether antibacterial resistance and/or AMR have increased or declined during the pandemic. However, the potential exacerbation of resistance phenomena due to the antibiotic overuse in COVID-19 patients was certainly one of the major concerns, and it was affected by very different parameters and conditions. A low prevalence of bacterial co-infections in the first 2–3 days of COVID-19 infections was ascertained, whereas higher rates were proved in severely ill patients, and those often were secondary bacterial infections. However, it is not a novelty that the irrational use of antibiotics, in low effective concentrations or non-specifically employed, represents one of the first causes of the overall resistance onset. Additionally, the lowering (or weakening) of immune system defenses, due to viral or bacterial infections, or a combination of both, very likely played a pivotal role in promoting the rise of resistance. Sustainable worldwide surveillance of social and clinical antibiotic consumption and resistance trends is crucial to anticipating subsequent changes and preventing antimicrobial shortages. It is extremely important to properly use the antibiotics to prevent complicated consequences, but the design and/or discovery of new compounds with antibacterial activity is enormously desirable and must be encouraged in order to be ready to face a possible “superbugs” bacterial pandemic. The lessons learned from the pandemic might represent a game changer in the fight against antibacterial resistance.

## Figures and Tables

**Table 1:** Names and Acronyms of Resistant Bacteria.

Name	Acronym	Resistant to:
Pan-drug-resistant	PDR	All antibiotics. even including colistin and tegacyclin
Difficult-to-treat drug-resistant	DTR	All first-line agents, including all β-lactams (carbapenems and β-lactamase inhibitor combinations) and fluoroquinolones
Extensively drug-resistant	XDR	All drugs except for colistin and tegacyclin
Multidrug-resistant	MDR	Three or even more antimicrobial agents
Carbapenems-resistant	CR	Carbapenems
Extended-spectrum β-lactamase	ESBL	Extended-spectrum β-lactamase, including penicillins, cephalosporins (also the third generation), and the monobactam aztreonam

**Table 2:** Names and Acronyms of Gram-negative Resistant Bacteria of Different Classes.

***Enterobacterales***	
Carbapenem-resistant *Enterobacterales*	CRE
Extended-spectrum β-lactamase (ESBL) producing *Enterobacterales*	EPE
Carbapenemase-producing *Enterobacterales*	CPE
***A. baumannii***	
Carbapenem-resistant *A. baumannii*	CR-Ab
Extensively drug-resistant *A. baumannii*	XDR-Ab
Multidrug-resistant *A. baumannii*	MDR-Ab
***P. aeruginosa***	
Difficult-to-treat drug-resistant *P. aeruginosa*	DTR-*Pa*
Carbapenem-resistant *P. aeruginosa*	CR-Pa
Extensively drug-resistant *P. aeruginosa*	XDR-Pa
***K. pneumoniae***	
Carbapenem-resistant *K. pneumoniae*	CR-Kp

**Table 3:** Therapies used for antimicrobial-resistant bacteria.

Structure	Name	Class
Doripenem	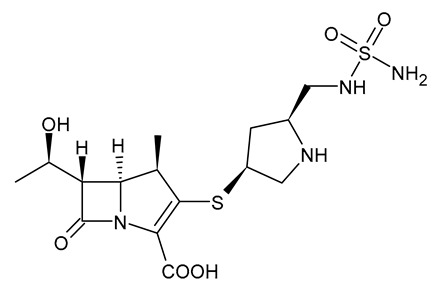	Carbapenem
Ertapenem	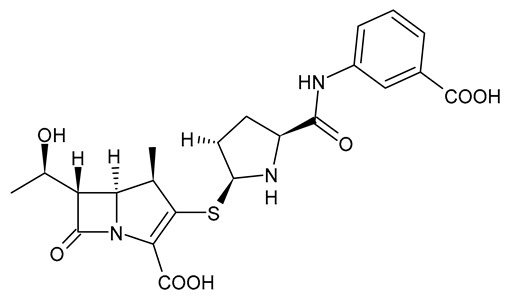	Carbapenem
Imipenem	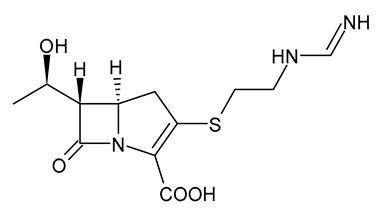	Carbapenem
Meropenem	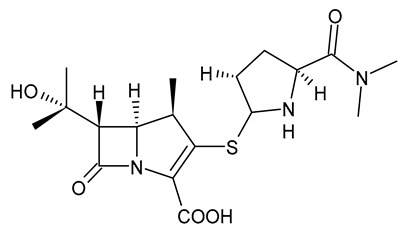	Carbapenem
Tigecycline	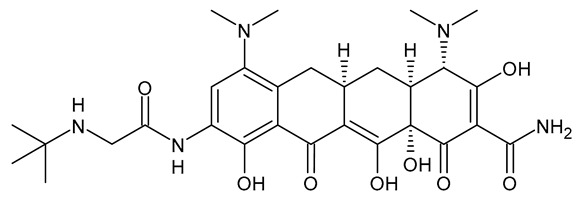	Glycylcycline Antibiotic
Ceftazidime	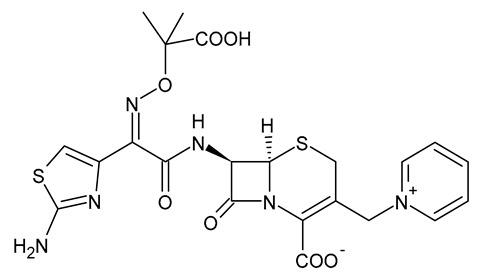	β-Lactam Antibiotic (Cephalosporin)
Cefiderocol	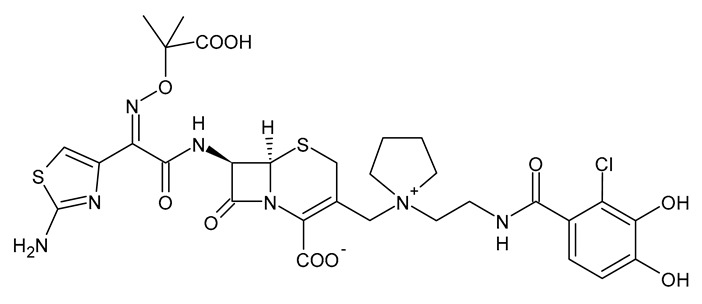	β-Lactam Antibiotic (Cephalosporin)
Ceftolozane	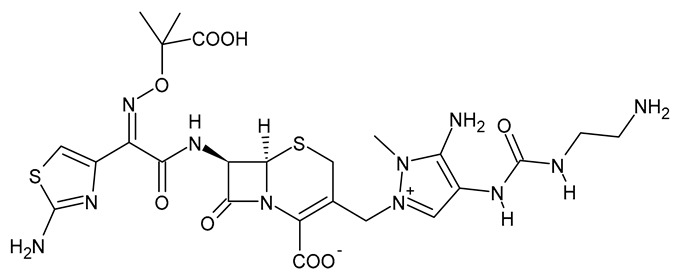	β-Lactam Antibiotic (Cephalosporin)
Avibactam	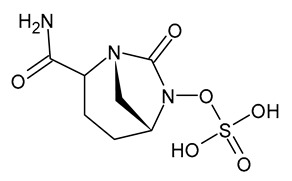	β-Lactamase Inhibitor
Tazobactam	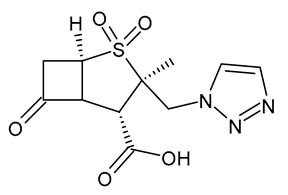	β-Lactamase Inhibitor
Vaborbactam	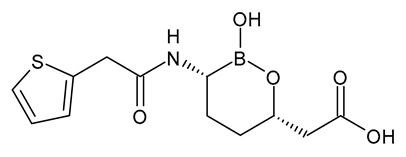	β-Lactamase Inhibitor
Durlobactam	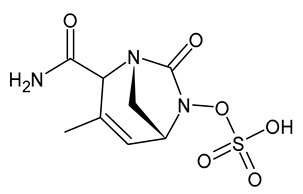	β-Lactamase Inhibitor
Plazomicin	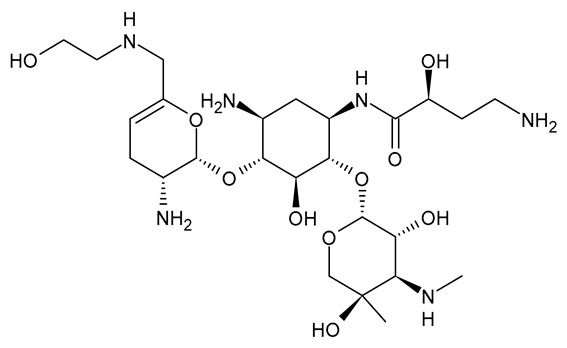	Aminoglycoside Antibiotic
Eravacycline	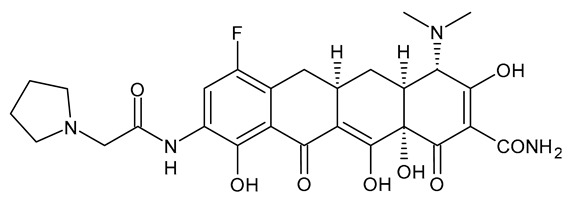	Fluorocycline Antibiotic

## Data Availability

Not applicable.

## References

[1] Strathdee S.A., Davies S.C., Marcelin J.R. (2020). Confronting antimicrobial resistance beyond the COVID-19 pandemic and the 2020 US election. Lancet.

[2] World Health Organization (2015). Global Action Plan on Antimicrobial Resistance. https://apps.who.int/iris/handle/10665/193736.

[3] World Health Organization (2022). One Health Joint Plan of Action (2022–2026): Working Together for the Health of Humans, Animals, Plants and the Environment.

[4] Jesudason T. (2022). A new One Health Joint Action Plan. Lancet Infect. Dis..

[5] Calvo-Villamañán A., San Millán Á., Carrilero L. (2022). Tackling AMR from a multidisciplinary perspective: A primer from education and psychology. Int. Microbiol..

[6] Adebisi Y.A., Ogunkola I.O. (2023). The global antimicrobial resistance response effort must not exclude marginalised populations. Trop. Med. Health.

[7] O’Neill J. (2014). Review on Antimicrobial Resistance: Tackling a Crisis for the Health and Wealth of Nations. https://amr-review.org/sites/default/files/AMR%20Review%20Paper%20-%20Tackling%20a%20crisis%20for%20the%20health%20and%20wealth%20of%20nations_1.pdf.

[8] Tackling Drug-Resistant Infections Globally: Final Report and Recommendations (2016). Review on Antimicrobial Resistance. https://amrreview.org/sites/default/files/160525_Final%20paper_with%20cover.pdf.

[9] Catalano A. (2020). COVID-19: Could irisin become the handyman myokine of the 21st century. Coronaviruses.

[10] Knight G.M., Glover R.E., McQuaid C.F., Olaru I.D., Gallandat K., Leclerc Q.J., Fuller N.M., Willcocks S.J., Hasan R., van Kleef E. (2021). Antimicrobial resistance and COVID-19: Intersections and implications. eLife.

[11] Toro-Alzate L., Hofstraat K., de Vries D.H. (2021). The pandemic beyond the pandemic: A Scoping review on the social relationships between COVID-19 and antimicrobial resistance. Int. J. Environ. Res. Public Health.

[12] Mahoney A.R., Safaee M.M., Wuest W.M., Furst A.L. (2021). The silent pandemic: Emergent antibacterial resistances following the global response to SARS-CoV-2. iScience.

[13] Rehman S. (2023). A parallel and silent emerging pandemic: Antimicrobial resistance (AMR) amid COVID-19 pandemic. J. Infect. Public Health.

[14] Votta M., Cardillo M. (2022). Civil society engagement in the fight against AMR: From the new National Plan in Italy to the AMR patient alliance at the European level. World J. Clin. Med. Images.

[15] Rawson T.M., Moore L.S.P., Castro-Sanchez E., Charani E., Davies F., Satta G., Ellington M.J., Holmes A.H. (2020). COVID-19 and the potential long-term impact on antimicrobial resistance. J. Antimicrob. Chemother..

[16] Getahun H., Smith I., Trivedi K., Paulin S., Balkhy H.H. (2020). Tackling antimicrobial resistance in the COVID-19 pandemic. Bull. World Health Organ..

[17] Monnet D.L., Harbarth S. (2020). Will coronavirus disease (COVID-19) have an impact on antimicrobial resistance?. Eurosurveillance.

[18] Founou R.C., Blocker A.J., Noubom M., Tsayem C., Choukem S.P., Van Dongen M., Founou L.L. (2021). The COVID-19 pandemic: A threat to antimicrobial resistance containment. Futur. Sci. OA.

[19] Ansari S., Hays J.P., Kemp A., Okechukwu R., Murugaiyan J., Ekwanzala M.D., Ruiz Alvarez M.J., Paul-Satyaseela M., Iwu C.D., Balleste-Delpierre C. (2021). The potential impact of the COVID-19 pandemic on global antimicrobial and biocide resistance: An AMR Insights global perspective. JAC Antimicrob. Resist..

[20] Langford B.J., So M., Raybardhan S., Leung V., Soucy J.-P.R., Westwood D., Daneman N., MacFadden D.R. (2021). Antibiotic prescribing in patients with COVID-19: Rapid review and meta-analysis. Clin. Microbiol. Infect..

[21] Sartelli M. (2021). COVID-19 impact on the understanding of infection prevention and control measures. Bangladesh J. Med. Sci..

[22] Ono A., Koizumi R., Tsuzuki S., Asai Y., Ishikane M., Kusama Y., Ohmagari N. (2022). Antimicrobial use fell substantially in Japan in 2020—The COVID-19 pandemic may have played a role. Int. J. Infect. Dis..

[23] Iacopetta D., Ceramella J., Catalano A., Saturnino C., Pellegrino M., Mariconda A., Longo P., Sinicropi M.S., Aquaro S. (2022). COVID-19 at a glance: An up-to-date overview on variants, drug design and therapies. Viruses.

[24] Sulayyim H.J.A., Ismail R., Hamid A.A., Ghafar N.A. (2022). Antibacterial resistance during COVID-19: A systematic review. Int. J. Environ. Res. Public Health.

[25] Ceramella J., Iacopetta D., Sinicropi M.S., Andreu I., Mariconda A., Saturnino C., Giuzio F., Longo P., Aquaro S., Catalano A. (2022). Drugs for COVID-19: An update. Molecules.

[26] Davis M.D., Lohm D., Flowers P., Whittaker A. (2023). The immune self, hygiene and performative virtue in general public narratives on antibiotics and antimicrobial resistance. Health.

[27] Banik G.R., Durayb B., King C., Rashid H. (2022). Antimicrobial resistance following prolonged use of hand hygiene products: A systematic review. Pharmacy.

[28] Sinicropi M.S., Iacopetta D., Ceramella J., Catalano A., Mariconda A., Pellegrino M., Saturnino C., Longo P., Aquaro S. (2022). Triclosan: A small molecule with controversial roles. Antibiotics.

[29] Iacopetta D., Catalano A., Ceramella J., Saturnino C., Salvagno L., Ielo I., Drommi D., Scali E., Plutino M.R., Rosace G. (2021). The different facets of triclocarban: A review. Molecules.

[30] Rizvi S.G., Ahammad S.Z. (2022). COVID-19 and antimicrobial resistance: A cross-study. Sci. Total Environ..

[31] Wicky P.-H., Niedermann M.S., Timsit J.-F. (2021). Ventilator-associated pneumonia in the era of COVID-19 pandemic: How common and what is the impact?. Crit. Care.

[32] Laxminarayan R., Van Boeckel T., Frost I., Kariuki S., Khan E.A., Limmathurotsakul D., Larsson D.G.J., Levy-Hara G., Mendelson M., Outterson K. (2020). The Lancet infectious diseases commission on antimicrobial resistance: 6 years later. Lancet Infect. Dis..

[33] Walia K., Mendelson M., Kang G., Venkatasubramanian R., Sinha R., Vijay S., Balaji Veeraraghavan B., Basnyat B., Rodrigues C., Bansal N. (2023). How can lessons from the COVID-19 pandemic enhance antimicrobial resistance surveillance and stewardship?. Lancet Infect. Dis..

[34] Hopman J., Allegranzi B., Mehtar S. (2020). Managing COVID-19 in low-and middle-income countries. JAMA.

[35] Lobie T.A., Roba A.A., Booth J.A., Kristiansen K.I., Aseffa A., Skarstad K., Bjørås M. (2021). Antimicrobial resistance: A challenge awaiting the post-COVID-19 era. Int. J. Infect. Dis..

[36] Ukuhor H.O. (2021). The interrelationships between antimicrobial resistance, COVID-19, past, and future pandemics. J. Infect. Public Health.

[37] Alejandro A.L., Bruce M., Leo C. (2022). Parents’ awareness of antimicrobial resistance: A qualitative study utilising the health belief model in Perth, Western Australia. Austral. New Zeal. J. Public Health.

[38] Ardebili A., Izanloo A., Rastegar M. (2023). Polymyxin combination therapy for multidrug-resistant, extensively-drug resistant, and difficult-to-treat drug-resistant Gram-negative infections: Is it superior to polymyxin monotherapy?. Exp. Rev. Anti-Infect. Ther..

[39] Catalano A., Iacopetta D., Ceramella J., Scumaci D., Giuzio F., Saturnino C., Aquaro S., Rosano C., Sinicropi M.S. (2022). Multidrug resistance (MDR): A widespread phenomenon in pharmacological therapies. Molecules.

[40] Quraini M.A., Jabri Z.A., Sami H., Mahindroo J., Taneja N., Muharrmi Z.A., Busaidi I.A., Rizvi M. (2023). Exploring synergistic combinations in extended and pan-drug resistant (XDR and PDR) whole genome sequenced *Acinetobacter Baumannii*. Microorg..

[41] Willyard C. (2017). The drug-resistant bacteria that pose the greatest health threats. Nature.

[42] Ma Y.X., Wang C.Y., Li Y.Y., Li J., Wan Q.Q., Chen J.H., Tay F.R., Niu L.N. (2019). Considerations and caveats in combating ESKAPE pathogens against nosocomial infections. Adv. Sci..

[43] Mulani M.S., Kamble E., Kumkar S.N., Tawre M.S., Pardesi K.R. (2019). Emerging strategies to combat ESKAPE pathogens in the era of antimicrobial resistance: A review. Front. Microbiol..

[44] Mancuso G., Midiri A., Gerace E., Biondo C. (2021). Bacterial antibacterial resistance: The most critical pathogens. Pathogens.

[45] Loyola-Cruz M.Á., Gonzalez-Avila L.U., Martínez-Trejo A., Saldaña-Padilla A., Hernández-Cortez C., Bello-López J.M., Castro-Escarpulli G. (2023). ESKAPE and beyond: The burden of coinfections in the COVID-19 pandemic. Pathogens.

[46] Langford B.J., Soucy J.R., Leung V., So M., Kwan A.T.H., Portnoff J.S., Bertagnolio S., Raybardhan S., MacFadden D.R., Daneman N. (2023). Antibacterial resistance associated with the COVID-19 pandemic: A systematic review and meta-analysis. Clin. Microbiol. Infect..

[47] Pérez Jorge G., Rodrigues Dos Santos Goes I.C., Gontijo M.T.P. (2022). Les misérables: A parallel between antimicrobial resistance and COVID-19 in underdeveloped and developing countries. Curr. Infect. Dis. Rep..

[48] Centers for Disease Control and Prevention (2019). Antibacterial Resistance Threats in the United States, 2019 (2019 AR Threats Report). https://www.cdc.gov/drugresistance/biggest-threats.html.

[49] Kumar M., Silori R., Mazumder P., Shrivastava V., Loge F., Barceló D., Mahlknecht J. (2023). Wars and pandemics: AMR accelerators of the 21^st^ century?. Environ. Sci. Technol. Lett..

[50] Magnano San Lio R., Favara G., Maugeri A., Barchitta M., Agodi A. (2023). How antimicrobial resistance is linked to climate change: An overview of two intertwined global challenges. Int. J. Environ. Res. Public Health.

[51] Kadri S.S., Adjemian J., Lai Y.L., Spaulding A.B., Ricotta E., Prevots D.R., Palmore T.N., Rhee C., Klompas M., Dekker J.P. (2018). Difficult-to-treat resistance in Gram-negative bacteremia at 173 US hospitals: Retrospective cohort analysis of prevalence, predictors, and outcome of resistance to all first-line agents. Clin. Infect. Dis..

[52] Coque T.M., Baquero F., Cantón R. (2008). Increasing prevalence of ESBL-producing Enterobacteriaceae in Europe. Eurosurveillance.

[53] Brolund A. (2014). Overview of ESBL-producing *Enterobacteriaceae* from a Nordic perspective. Infect. Ecol. Epidemiol..

[54] Lee Y.L., Chen H.M., Hii M., Hsueh P.R. (2022). Carbapenemase-producing *Enterobacterales* infections: Recent advances in diagnosis and treatment. Int. J. Antimicrob. Agents.

[55] Tamma P.D., Aitken S.L., Bonomo R.A., Mathers A.J., van Duin D., Clancy C.J. (2021). Infectious Diseases Society of America Guidance on the treatment of Extended-Spectrum β-lactamase producing Enterobacterales (ESBL-E), carbapenem-resistant Enterobacterales (CRE), and *Pseudomonas aeruginosa* with Difficult-to-Treat Resistance (DTR-P. aeruginosa). Clin. Infect. Dis..

[56] Tacconelli E., Carrara E., Savoldi A., Harbarth S., Mendelson M., Monnet D.L., Pulcini C., Kahlmeter G., Kluytmans J., Carmeli Y. (2018). Discovery, research, and development of new antibiotics: The WHO Priority list of antibiotic-resistant bacteria and tuberculosis. Lancet Infect. Dis..

[57] Paul M., Carrara E., Retamar P., Tängdén T., Bitterman R., Bonomo R.A., de Waele J., Daikos G.L., Akova M., Harbarth S. (2022). European Society of clinical microbiology and infectious diseases (ESCMID) guidelines for the treatment of infections caused by Multidrug-resistant Gram-negative bacilli (endorsed by ESICM-European Society of intensive care Medicine). Clin. Microbiol. Infect..

[58] Semenec L., Cain A.K., Dawson C.J., Liu Q., Dinh H., Lott H., Penesyan A., Maharjan R., Short F.L., Hassan K.A. (2023). Cross-protection and cross-feeding between *Klebsiella pneumoniae* and *Acinetobacter baumannii* promotes their co-existence. Nat. Commun..

[59] Tacconelli E., Pezzani M.D. (2019). Public health burden of antimicrobial resistance in Europe. Lancet Infect. Dis..

[60] Durán-Manuel E.M., Cruz-Cruz C., Ibáñez-Cervantes G., Bravata-Alcantará J.C., Sosa-Hernández O., Delgado-Balbuena L., León-García G., Cortés-Ortíz I.A., Cureño-Díaz M.A., Castro-Escarpulli G. (2021). Clonal dispersion of *Acinetobacter baumannii* in an intensive care unit designed to patients COVID-19. J. Infect. Dev. Ctries.

[61] McAdam A.J. (2020). Enterobacteriaceae? Enterobacterales? What should we call enteric gram-negative bacilli? A micro-comic strip. J. Clin. Microbiol..

[62] Davin-Regli A., Pagès J.M. (2015). *Enterobacter aerogenes* and *Enterobacter cloacae*; versatile bacterial pathogens confronting antibiotic treatment. Front. Microbiol..

[63] Davin-Regli A., Masi M., Bialek S., Nicolas-Chanoine M.H., Pagès J.-M., Li X.-Z., Elkins C.A., Zgurskaya H.I. (2016). Antimicrobial resistance and drug efflux pumps in Enterobacter and *Klebsiella*. Efflux-Mediated Drug Resistance in Bacteria: Mechanisms, Regulation and Clinical Implications.

[64] Rangel K., Chagas T.P.G., De-Simone S.G. (2021). *Acinetobacter baumannii* infections in times of COVID-19 pandemic. Pathogens.

[65] Baumann P., Doudoroff M., Stanier R.Y. (1968). A study of the *Moraxella* group. II. Oxidative-negative species (genus *Acinetobacter*). J. Bacteriol..

[66] Karabay O., Ekşi F., Yıldırım M.S. (2022). Investigation of antibiotic resistance profiles and carbapenemase resistance genes in *Acinetobacter baumannii* strains isolated from clinical samples. Eur. J. Ther..

[67] Mancuso G., De Gaetano S., Midiri A., Zummo S., Biondo C. (2023). The Challenge of overcoming antibiotic resistance in carbapenem-resistant Gram-negative bacteria: “Attack on Titan”. Microorganisms.

[68] Murray C.K., Roop S.A., Hospenthal D.R., Dooley D.P., Wenner K., Hammock J., Taufen N., Gourdine E. (2006). Bacteriology of war wounds at the time of injury. Mil. Med..

[69] Schultze T., Hogardt M., Velázquez E.S., Hack D., Besier S., Wichelhaus T.A., Rochwalsky U., Kempf V.A., Reinheimer C. (2023). Molecular surveillance of multidrug-resistant Gram-negative bacteria in Ukrainian patients, Germany, March to June 2022. Euro. Surveill..

[70] Whiteway C., Breine A., Philippe C., Van der Henst C. (2022). *Acinetobacter* *baumannii*. Trends Microbiol..

[71] Nocera F.P., De Martino L. (2023). Editorial on the research topic of the Special Issue “Current Status of *Acinetobacter* infections”. Pathogens.

[72] Higgins P.G., Hagen R.M., Podbielski A., Frickmann H., Warnke P. (2020). Molecular epidemiology of carbapenem-resistant *Acinetobacter baumannii* isolated from war-injured patients from the Eastern Ukraine. Antibiotics.

[73] Serapide F., Quirino A., Scaglione V., Morrone H.L., Longhini F., Bruni A., Garofalo E., Matera G., Marascio N., Scarlata G.G.M. (2022). Is the pendulum of antimicrobial drug resistance swinging back after COVID-19?. Microorganisms.

[74] Pascale R., Bussini L., Gaibani P., Bovo F., Fornaro G., Lombardo D., Ambretti S., Pensalfine G., Appolloni L., Bartoletti M. (2022). Carbapenem-resistant bacteria in an intensive care unit during the Coronavirus Disease 2019 (COVID-19) pandemic: A multicenter before-and-after cross-sectional study. Infect. Control Hosp. Epidemiol..

[75] Russo A., Gavaruzzi F., Ceccarelli G., Borrazzo C., Oliva A., Alessandri F., Magnanimi E., Pugliese F., Venditti M. (2022). Multidrug-resistant *Acinetobacter baumannii* infections in COVID-19 patients hospitalized in intensive care unit. Infection.

[76] Nutman A., Lerner A., Schwartz D., Carmeli Y. (2016). Evaluation of carriage and environmental contamination by carbapenem-resistant *Acinetobacter baumannii*. Clin. Microbiol. Infect..

[77] Doidge M., Allworth A.M., Woods M., Marshall P., Terry M., O’Brien K., Goh H.M., George N., Nimmo G.R., Schembri M.A. (2010). Control of an outbreak of carbapenem-resistant *Acinetobacter baumannii* in Australia after introduction of environmental cleaning with a commercial oxidizing disinfectant. Infect. Control Hosp. Epidemiol..

[78] Perez S., Innes G.K., Walters M.S., Mehr J., Arias J., Greeley R., Chew D. (2020). Increase in hospital-acquired carbapenem-resistant *Acinetobacter baumannii* infection and colonization in an acute care hospital during a surge in COVID-19 admissions—New Jersey, February–July 2020. Morb. Mort. Weekly Rep..

[79] Zhang G., Hu C., Luo L., Fang F., Chen Y., Li J., Peng Z., Pan H. (2020). Clinical features and outcomes of 221 patients with COVID-19 in Wuhan, China. J. Clin. Virol..

[80] Cafiso V., Stracquadanio S., Lo Verde F., Dovere V., Zega A., Pigola G., Aranda J., Stefani S. (2020). COL^R^ *Acinetobacter baumannii* sRNA signatures: Computational comparative identification and biological targets. Front. Microbiol..

[81] Bagley S.T. (1985). Habitat association of *Klebsiella* species. Infect. Control.

[82] Rock C., Thom K.A., Masnick M., Johnson J.K., Harris A.D., Morgan D.J. (2014). Frequency of *Klebsiella pneumoniae* carbapenemase (KPC)-producing and non-KPC-producing *Klebsiella* species contamination of healthcare workers and the environment. Infect. Control Hosp. Epidemiol..

[83] Friedlander C. (1882). Uber die scizomyceten bei der acuten fibrosen pneumonie. Arch. Pathol. Anat. Physiol. Klin. Med..

[84] Merino S., Camprubi S., Alberti S., Benedi V.J., Tomas J.M. (1992). Mechanisms of *Klebsiella pneumoniae* resistance to complement-mediated killing. Infect. Immun..

[85] Paczosa M.K., Mecsas J. (2016). *Klebsiella pneumoniae*: Going on the offense with a strong defense. Microbiol. Mol. Biol. Rev..

[86] Martin R.M., Bachman M.A. (2018). Colonization, infection, and the accessory genome of *Klebsiella pneumoniae*. Front. Cell. Infect. Microbiol..

[87] Podschun R., Ullmann U. (1998). *Klebsiella* spp. as nosocomial pathogens: Epidemiology, taxonomy, typing methods, and pathogenicity factors. Clin. Microbiol. Rev..

[88] Yuling Z., Zhao Y., Liu C., Chen Z., Zhou D. (2014). Molecular pathogenesis of *Klebsiella pneumoniae*. Future Microbiol..

[89] Kotb S., Lyman M., Ismail G., El Fattah M.A., Girgis S.A., Etman A., Hafez S., El-Kholy J., Zaki M.E.S., Rashed H.-A.G. (2020). Epidemiology of carbapenem-resistant Enterobacteriaceae in Egyptian intensive care units using National Healthcare–associated Infections Surveillance Data, 2011–2017. Antimicrob. Resist. Infect. Control.

[90] Mędrzycka-Dąbrowska W., Lange S., Zorena K., Dąbrowski S., Ozga D., Tomaszek L. (2021). Carbapenem-resistant *Klebsiella pneumoniae* infections in ICU COVID-19 patients—A scoping review. J. Clin. Med..

[91] Westblade L.F., Simon M.S., Satlin M.J. (2021). Bacterial coinfections in Coronavirus Disease 2019. Trends Microbiol..

[92] Hosoda T., Harada S., Okamoto K., Ishino S., Kaneko M., Suzuki M., Ito R., Mizoguchi M. (2021). COVID-19 and fatal sepsis caused by hypervirulent *Klebsiella pneumoniae*, Japan, 2020. Emerg. Infect. Dis..

[93] Arteaga-Livias K., Pinzas-Acosta K., Perez-Abad L., Panduro-Correa V., Rabaan A.A., Pecho-Silva S., Dámaso-Mata B. (2022). A multidrug-resistant *Klebsiella pneumoniae* outbreak in a Peruvian hospital: Another threat from the COVID-19 pandemic. Infect. Control Hosp. Epidemiol..

[94] Miftode I.L., Leca D., Miftode R.S., Roşu F., Plesca C., Loghin I., Timpau A.S., Mitu I., Mitituc T., Dorneanu O. (2023). The clash of the titans: COVID-19, carbapenem-resistant Enterobacterales, and first *mcr*-1-mediated colistin resistance in humans in Romania. Antibiotics.

[95] Chang D., Sharma L., Cruz C.S.D., Zhang D. (2021). Clinical epidemiology, risk factors, and control strategies of *Klebsiella pneumoniae* infection. Front. Microbiol..

[96] Russo T.A., Marr C.M. (2019). Hypervirulent *Klebsiella pneumoniae*. Clin. Microbiol. Rev..

[97] Catalan-Najera J.C., Garza-Ramos U., Barrios-Camacho H. (2017). Hypervirulence and hypermucoviscosity: Two different but complementary *Klebsiella* spp. phenotypes?. Virulence.

[98] Cunningham E.T., Zierhut M. (2022). Hypervirulent, multidrug resistant *Klebsiella pneumoniae*–Emergence of a superbug of concern for eye care providers. Ocular Immunol. Inflamm..

[99] Siu L.K., Yeh K.M., Lin J.C., Fung C.P., Chang F.Y. (2012). *Klebsiella pneumoniae* liver abscess: A new invasive syndrome. Lancet Infect. Dis..

[100] Choby J.E., Howard-Anderson J., Weiss D.S. (2020). Hypervirulent *Klebsiella pneumoniae*–clinical and molecular perspectives. J. Int. Med..

[101] Lederman E.R., Crum N.F. (2005). Pyogenic liver abscess with a focus on *Klebsiella pneumoniae* as a primary pathogen: An emerging disease with unique clinical characteristics. Am. J. Gastroenterol..

[102] Struve C., Roe C.C., Stegger M., Stahlhut S.G., Hansen D.S., Engelthaler D.M., Andersen P.S., Driebe E.M., Keim P., Krogfelt K.A. (2015). Mapping the evolution of hypervirulent *Kleb*. Pneumoniae Mbio..

[103] Shankar C., Veeraraghavan B., Nabarro L.E.B., Ravi R., Ragupathi N.K.D., Rupali P. (2018). Whole genome analysis of hypervirulent *Klebsiella pneumoniae* isolates from community and hospital acquired bloodstream infection. BMC Microbiol..

[104] Liu C., Du P., Xiao N., Ji F., Russo T.A., Guo J. (2020). Hypervirulent *Klebsiella pneumoniae* is emerging as an increasingly prevalent *K. pneumoniae* pathotype responsible for nosocomial and healthcare-associated infections in Beijing, China. Virulence.

[105] Rafat C., Messika J., Barnaud G., Dufour N., Magdoud F., Billard-Pomarès T., Gaudry S., Dreyfuss D., Branger C., Decré D. (2018). Hypervirulent *Klebsiella pneumoniae*, a 5-year study in a French ICU. J. Med. Microbiol..

[106] Turton J.F., Payne Z., Coward A., Hopkins K.L., Turton J.A., Doumith M., Woodford N. (2018). Virulence genes in isolates of Klebsiella pneumoniae from the UK during 2016, including among carbapenemase gene-positive hypervirulent K1-ST23 and ‘non-hypervirulent’ types ST147, ST15 and ST383. J. Med. Microbiol..

[107] Odouard C., Ong D., Shah P.R., Gin T., Allen P.J., Downie J., Lim L., McCluskey P. (2016). Rising trends of endogenous *Klebsiella pneumoniae* endophthalmitis in Australia. Clin. Exp. Ophthalmol..

[108] Mgbemena O., Serota D.P., Kumar S., Wozniak J.E., Weiss D.S., Kempker R.R. (2017). Peculiar purulence: Hypervirulent *Klebsiella pneumoniae* causing pyomyositis. Int. J. Infect. Dis..

[109] Kamau E., Ranson E.L., Tsan A.T., Bergmann-Leitner E.S., Garner O.B., Yang S. (2022). Clinical and genomic characterization of hypervirulent *Klebsiella pneumoniae* (hvKp) infections via passive surveillance in Southern California, 2020–2022. Front. Microbiol..

[110] Lan P., Jiang Y., Zhou J., Yu Y. (2021). A global perspective on the convergence of hypervirulence and carbapenem resistance in *Klebsiella pneumoniae*. J. Glob. Antimicrob. Resist..

[111] Sleiman A., Awada B., Mocadie M., Sherri N., Haraoui L.P., Baby V., Araj G.F., Kanj S.S., Rizk N., Matar G.M. (2021). An unequivocal superbug: PDR *Klebsiella pneumoniae* with an arsenal of resistance and virulence factor genes. J. Infect. Dev. Ctries..

[112] Aschtgen M.S., Henriques-Normark B., Normark S. (2020). The rise of hyper-virulence. J. Intern. Med..

[113] Antibacterial Resistance Threats in the United States (2019). Centers for Disease Control. https://www.cdc.gov/drugresistance/pdf/threats-report/2019-ar-threats-report-508.pdf.

[114] World Health Organization Global Priority List of Antibacterial Resistance Bacteria to Guide Research, Discovery, and Development of New Antibiotics. https://www.who.int/medicines/publications/WHO-PPL-Short_Summary_25Feb-ET_NM_WHO.pdf.

[115] Nordmann P., Poirel L. (2002). Emerging carbapenemases in Gram-negative aerobes. Clin. Microbiol. Infect..

[116] Brink A.J. (2019). Epidemiology of carbapenem-resistant Gram-negative infections globally. Curr. Opin. Infect. Dis..

[117] Katip W., Meechoui M., Thawornwittayakom P., Chinwong D., Oberdorfer P. (2019). Efficacy and safety of high loading dose of colistin in multidrug-resistant *Acinetobacter baumannii*: A prospective cohort study. J. Intensive Care Med..

[118] Giammanco A., Calà C., Fasciana T., Dowzicky M.J. (2017). Global assessment of the activity of tigecycline against multidrug-resistant Gram-negative pathogens between 2004 and 2014 as part of the tigecycline evaluation and surveillance trial. Msphere.

[119] Novović K., Jovčić B. (2023). Colistin resistance in *Acinetobacter baumannii*: Molecular mechanisms and epidemiology. Antibiotics.

[120] Pankey G.A. (2005). Tigecycline. J. Antimicrob. Chemother..

[121] Mantzana P., Protonotariou E., Kassomenaki A., Meletis G., Tychala A., Keskilidou E., Arhonti M., Katsanou C., Daviti A., Vasilaki O. (2023). *In vitro* synergistic activity of antimicrobial combinations against carbapenem-and colistin-resistant *Acinetobacter baumannii* and *Klebsiella pneumoniae*. Antibiotics.

[122] Collalto D., Fortuna A., Visca P., Imperi F., Rampioni G., Leoni L. (2023). Synergistic activity of colistin in combination with clofoctol against colistin resistant gram-negative pathogens. Microbiol. Spectr..

[123] Liu Y.Y., Qin Z.H., Yue H.Y., Bergen P.J., Deng L.M., He W.Y., Zeng Z.L., Peng X.F., Liu J.H. (2023). Synergistic effects of capric acid and colistin against colistin-susceptible and colistin-resistant Enterobacterales. Antibiotics.

[124] Zhang Y., Han Y., Wang L., Kong J., Pan W., Zhang X., Chen L., Yao Z., Cao J. (2023). Flufenamic acid, a promising agent for the sensitization of colistin-resistant Gram-negative bacteria to colistin. Microbiol. Spectr..

[125] Burastero G.J., Orlando G., Santoro A., Menozzi M., Franceschini E., Bedini A., Cervo A., Faltoni M., Bacca E., Biagioni E. (2022). Ceftazidime/avibactam in ventilator-associated pneumonia due to difficult-to-treat non-fermenter gram-negative bacteria in COVID-19 patients: A case series and review of the literature. Antibiotics.

[126] Chi Y., Xu J., Bai N., Liang B., Cai Y. (2023). The efficacy and safety of ceftolozane-tazobactam in the treatment of GNB infections: A meta-analysis of clinical studies. Exp. Rev. Anti-infect. Ther..

[127] Khankhel Z.S., Dillon R.J., Thosar M., Bruno C., Puzniak L. (2022). Ceftolozane/tazobactam for the treatment of bacteremia: A systematic literature review (SLR). Ann. Clin. Microbiol. Antimicrob..

[128] Huang W., Hamouche J.E., Wang G., Smith M., Yin C., Dhand A., Dimitrova N., Fallon J.T. (2020). Integrated genome-wide analysis of an isogenic pair of *Pseudomonas aeruginosa* clinical isolates with differential antimicrobial resistance to ceftolozane/tazobactam, ceftazidime/avibactam, and piperacillin/tazobactam. Int. J. Mol. Sci..

[129] Novelli A., Del Giacomo P., Rossolini G.M., Tumbarello M. (2020). Meropenem/vaborbactam: A next generation β-lactam β-lactamase inhibitor combination. Expert Rev. Anti. Infect. Ther..

[130] O’Donnell J.N., Lodise T.P. (2022). New perspectives on antimicrobial agents: Imipenem-relebactam. Antimicrob. Ag. Chemother..

[131] Naik J., Dillon R., Massello M., Ralph L., Yang Z. (2023). Cost–effectiveness of imipenem/cilastatin/relebactam for hospital-acquired and ventilator-associated bacterial pneumonia. J. Compar. Eff. Res..

[132] Shaeer K.M., Zmarlicka M.T., Chahine E.B., Piccicacco N., Cho J.C. (2019). Plazomicin: A next-generation aminoglycoside. Pharmacother. J. Hum. Pharmacol. Drug Ther..

[133] Lee Y.R., Burton C.E. (2019). Eravacycline, a newly approved fluorocycline. Eur. J. Clin. Microbiol. Infect. Dis..

[134] Ong’uti S., Czech M., Robilotti E., Holubar M. (2022). Cefiderocol: A new cephalosporin stratagem against multidrug-resistant Gram-negative bacteria. Clin. Infect. Dis..

[135] Doi Y. (2019). Treatment options for carbapenem-resistant Gram-negative bacterial infections. Clin. Infect. Dis..

[136] Gaibani P., Giani T., Bovo F., Lombardo D., Amadesi S., Lazzarotto T., Coppi M., Rossolini G.M., Ambretti S. (2022). resistance to ceftazidime/avibactam, meropenem/vaborbactam and imipenem/relebactam in Gram-negative MDR bacilli: Molecular mechanisms and susceptibility testing. Antibiotics.

[137] El-Ghali A., Kunz Coyne A.J., Caniff K., Bleick C., Rybak M.J. (2023). Sulbactam-durlobactam: A Novel β-lactam-β-lactamase inhibitor combination targeting carbapenem-resistant *Acinetobacter baumannii* infections. Pharmacother. J. Human Pharmacol. Drug Ther..

[138] Murugaiyan J., Kumar P.A., Rao G.S., Iskandar K., Hawser S., Hays J.P., Mohsen Y., Adukkadukkam S., Awuah W.A., Jose R.A.M. (2022). Progress in alternative strategies to combat antimicrobial resistance: Focus on antibiotics. Antibiotics.

[139] Wu N., Chen L.-K., Zhu T. (2022). Phage therapy for secondary bacterial infections with COVID-19. Curr. Opin. Virol..

[140] Gholizadeh P., Kose S., Dao S., Ganbarov K., Tanomand A., Dal T., Aghazadeh M., Ghotaslou R., Ahangarzadeh Rezaee M., Yousefi B. (2020). How CRISPR-Cas system could be used to combat antimicrobial resistance. Infect. Drug Resist..

[141] Hu Y., Lyu Y., Jia X., Yue C., Deng S., Zhang X. (2023). Non-antibiotic prevention and treatment against *Acinetobacter baumannii* infection: Are vaccines and adjuvants effective strategies?. Front. Microbiol..

[142] Ibrahim S., Al-Saryi N., Al-Kadmy I.M.S., Aziz S.N. (2021). Multidrug-resistant *Acinetobacter baumannii* as an emerging concern in hospitals. Mol. Biol. Rep..

[143] Hujer A.M., Higgins P.G., Rudin S.D., Buser G.L., Marshall S.H., Xanthopoulou K., Seifert H., Rojas L.J., Domitrovic T.N., Cassidy P.M. (2017). Nosocomial outbreak of extensively drug-resistant *Acinetobacter baumannii* isolates containing blaOXA-237 carried on a plasmid. Antimicrob. Agents Chemother..

[144] Russell C.D., Fairfield C.J., Drake T.M., Turtle L., Seaton R.A., Wootton D.G., Sigfrid L., Harrison E.M., Docherty A.B., de Silva T.I. (2021). Co-infections, secondary infections, and antimicrobial use in patients hospitalised with COVID-19 during the first pandemic wave from the ISARIC WHO CCP-UK study: A multicentre, prospective cohort study. Lancet Microbe.

[145] Segala F.V., Bavaro D.F., Di Gennaro F., Salvati F., Marotta C., Saracino A., Murri R., Fantoni M. (2021). Impact of SARS-CoV-2 epidemic on antimicrobial resistance: A literature review. Viruses.

[146] Cox M.J., Loman N., Bogaert D., O’Grady J. (2020). Co-infections: Potentially lethal and unexplored in COVID-19. Lancet Microbe.

[147] Kariyawasam R.M., Julien D.A., Jelinski D.C., Larose S.L., Rennert-May E., Conly J.M., Dingle T.C., Chen J.Z., Tyrrell G.J., Ronksley P.E. (2022). Antimicrobial resistance (AMR) in COVID-19 patients: A systematic review and meta-analysis (November 2019–June 2021). Antimicrob. Resist. Infect. Control..

[148] Silvester R., Madhavan A., Kokkat A., Parolla A., Adarsh B.M., Harikrishnan M., Abdulla M.H. (2022). Global surveillance of antimicrobial resistance and hypervirulence in *Klebsiella pneumoniae* from LMICs: An in-silico approach. Sci. Total Environ..

[149] Brandi N., Ciccarese F., Balacchi C., Rimondi M.R., Modolon C., Sportoletti C., Capozzi C., Renzulli M., Paccapelo A., Castelli A. (2022). Co-infections and superinfections in COVID-19 critically Ill patients are associated with CT imaging abnormalities and the worst outcomes. Diagnostics.

[150] Langford B.J., So M., Leung V., Raybardhan S., Lo J., Kan T., Leung F., Westwood D., Daneman N., MacFadden D.R. (2022). Predictors and microbiology of respiratory and bloodstream bacterial infection in patients with COVID-19: Living rapid review update and meta-regression. Clin. Microbiol. Infect..

[151] Langford B.J., So M., Raybardhan S., Leung V., Westwood D., MacFadden D.R., Soucy J.P.R., Daneman N. (2020). Bacterial coinfection and secondary infection in patients with COVID-19: A living rapid review and meta-analysis. Clin. Microb. Infect..

[152] Fu Y., Yang Q.X.M., Kong H., Chen H., Fu Y., Yao Y., Zhou H., Zhou J. (2020). Secondary bacterial infections in critically ill patients with coronavirus disease 2019. Open Forum Infect. Dis..

[153] Lansbury L., Lim B., Baskaran V., Lim W.S. (2020). Coinfections in people with COVID-19: A systematic review and meta-analysis. J. Infect..

[154] Lai C.C., Wang C.Y., Hsueh P.R. (2020). Co-infections among patients with COVID-19: The need for combination therapy with non-anti-SARS-CoV-2 agents?. J. Microbiol. Immunol. Infect..

[155] Lavrinenko A., Kolesnichenko S., Kadyrova I., Turmukhambetova A., Akhmaltdinova L., Klyuyev D. (2023). Bacterial co-infections and antimicrobial resistance in patients hospitalized with suspected or confirmed COVID-19 pneumonia in Kazakhstan. Pathogens.

[156] Iacovelli A., Oliva A., Siccardi G., Tramontano A., Pellegrino D., Mastroianni C.M., Venditti M., Palange P. (2023). Risk factors and effect on mortality of superinfections in a newly established COVID-19 respiratory sub-intensive care unit at University Hospital in Rome. BMC Pulm. Med..

[157] Floridia M., Giuliano M., Monaco M., Palmieri L., Lo Noce C., Palamara A.T., Pantosti A., Brusaferro S., Onder G. (2022). Microbiologically confirmed infections and antibiotic-resistance in a national surveillance study of hospitalised patients who died with COVID-19, Italy 2020–2021. Antimicrob. Res. Infect. Control.

[158] Traglia G.M., Pasteran F., Escalante J., Nishimura B., Tuttobene M.R., Subils T., Nuñez M.R., Rivollier M.G., Corso A., Tolmasky M.E. (2023). Genomic comparative analysis of two multi-drug resistance (MDR) *Acinetobacter baumannii* clinical strains assigned to international clonal lineage II recovered pre-and post-COVID-19 pandemic. Biology.

[159] Saleh Ahmed M., Abdulrahman Z.F.A., Taha Z.M.A. (2023). Risk factors of clonally related, multi, and extensively drug-resistant *Acinetobacter baumannii* in severely Ill COVID-19 patients. Canad. J. Infect. Dis. Med. Microbiol..

[160] Boorgula S.Y., Yelamanchili S., Kottapalli P., Naga M.D. (2022). An update on secondary bacterial and fungal infections and their antimicrobial resistance pattern (AMR) in COVID-19 confirmed patients at a tertiary care hospital. J. Lab. Physicians.

[161] Tomczyk S., Taylor A., Brown A., de Kraker M.E.A., El-Saed A., Alshamrani M., Hendriksen R.S., Jacob M., Löfmark S., Perovic O. (2021). Impact of the COVID-19 pandemic on the surveillance, prevention and control of antimicrobial resistance: A global survey. J. Antimicrob. Chemother..

[162] Murray A.K. (2020). The novel coronavirus COVID-19 outbreak: Global implications for antimicrobial resistance. Front. Microbiol..

[163] Shrestha P., He S., Legido-Quigley H. (2022). Antimicrobial resistance research collaborations in Asia: Challenges and opportunities to equitable partnerships. Antibiotics.

[164] Daria S., Islam M.R. (2022). Indiscriminate use of antibiotics for COVID-19 treatment in South Asian Countries is a threat for future pandemics due to antibacterial resistance. Clin. Pathol..

[165] Lambraki I., Chadag M., Cousins M., Graells T., Léger A., Henriksson P., Troell M., Harbarth S.J., Wernli D., Søgaard Jørgensen P. (2022). Antimicrobial resistance in South East Asia: A participatory systems modelling approach. Int. J. Infect. Dis..

[166] Vijay S., Bansal N., Rao B.K., Veeraraghavan B., Rodrigues C., Wattal C., Goyal J.P., Tadepalli K., Mathur P., Venkateswaran R. (2021). Secondary infections in hospitalized COVID-19 patients: Indian experience. Infect. Drug Resist..

[167] Saini V., Jain C., Singh N., Alsulimani A., Gupta C., Dar S., Haque S., Das S. (2021). Paradigm shift in antimicrobial resistance pattern of bacterial isolates during the COVID-19 pandemic. Antibiotics.

[168] Ahmed N., Khan M., Saleem W., Karobari M.I., Mohamed R.N., Heboyan A., Rabaan A.A., Mutair A.A., Alhumaid S., Alsadiq S.A. (2022). Evaluation of bi-lateral co-infections and antibacterial resistance rates among COVID-19 patients. Antibiotics.

[169] OECD, ECDC (2019). Antimicrobial Resistance—Tackling the Burden in the European Union—Briefing Note for EU/EEA Countries. https://www.oecd.org/health/health-systems/AMR-Tackling-the-Burden-in-the-EU-OECD-ECDC-Briefing-Note-2019.pdf.

[170] Khaznadar O., Khaznadar F., Petrovic A., Kuna L., Loncar A., Kolaric T.O., Mihaljevic V., Tabll A.A., Smolic R., Smolic M. (2023). Antimicrobial resistance and antimicrobial stewardship: Before, during and after the COVID-19 pandemic. Microbiol. Res..

[171] World Health Organization (2022). Antimicrobial Resistance Surveillance in Europe 2022–2020 Data.

[172] Gysin M., Acevedo C.T., Haldimann K., Bodendoerfer E., Imkamp F., Bulut K., Buehler P.K., Brugger S.D., Becker K., Hobbie S.N. (2021). Antimicrobial susceptibility patterns of respiratory Gram-negative bacterial isolates from COVID-19 patients in Switzerland. Ann. Clin. Microbiol. Antimicrob..

[173] Cogliati Dezza F., Arcari G., Alessi F., Valeri S., Curtolo A., Sacco F., Ceccarelli G., Raponi G., Alessandri F., Mastroianni C.M. (2022). Clinical impact of COVID-19 on multi-drug-resistant Gram-negative bacilli bloodstream infections in an intensive care unit setting: Two pandemics compared. Antibiotics.

[174] Falcone M., Tiseo G., Arcari G., Leonildi A., Giordano C., Tempini S., Bibbolino G., Mozzo R., Barnini S., Carattoli A. (2022). Spread of hypervirulent multidrug-resistant ST147 *Klebsiella pneumoniae* in patients with severe COVID-19: An observational study from Italy, 2020–2021. J. Antimicrob. Chemother..

[175] Mbow M., Lell B., Jochems S.P., Cisse B., Mboup S., Dewals B.G., Jaye A., Dieye A., Yazdanbakhsh M. (2020). COVID-19 in Africa: Dampening the storm?. Science.

[176] Gutema G., Homa G. (2022). Cropping up crisis at the nexus between COVID-19 and antimicrobial resistance (AMR) in Africa: A scoping review and synthesis of early evidence. Cureus.

[177] Harant A. (2022). Assessing transparency and accountability of national action plans on antimicrobial resistance in 15 african countries. Antimicrob. Resist. Infect. Control.

[178] Mutua J.M., Njeru J.M., Musyoki A.M. (2022). Multidrug resistant bacterial infections in severely ill COVID-19 patients admitted in a national referral and teaching hospital, Kenya. BMC Infect. Dis..

[179] Adebisi Y.A., Jimoh N.D., Ogunkola I.O., Uwizeyimana T., Olayemi A.H., Ukor N.A., Lucero-Prisno D.E. (2021). The use of antibiotics in COVID-19 management: A rapid review of national treatment guidelines in 10 African countries. Trop. Med. Health.

[180] Tull F., Bamert R.S., Smith L., Goodwin D., Lambert K. (2023). Identifying and prioritising behaviours to slow antimicrobial resistance. Antibiotics.

[181] Merlino J., Siarakas S. (2022). Antibiotic prescribing and antimicrobial resistance from an Australian perspective. Microb. Drug Resist..

[182] Australian Government Department of Health (2021). 2019 Influenza Season in Australia. A Summary from the National Influenza Surveillance Committee. https://www1.health.gov.au/internet/main/publishing.nsf/Content/7610377A5BEB1D25CA25874B007D9DD2/%24File/2019-Influenza-Season-Summary.pdf.

[183] Basseal J.M., Bennett C.M., Collignon P., Currie B.J., Durrheim D.N., Leask J., McBryde E.S., McIntyre P., Russell F.M., Smith D.W. (2022). Key lessons from the COVID-19 public health response in Australia. Lancet Reg. Health—West. Pac..

[184] Lindgren M., Jorgensen B. (2023). Podcasting and constructive journalism in health stories about antimicrobial resistance (AMR). Media Int. Australia.

[185] Bauer K.A., Puzniak L.A., Yu K.C., Klinker K.P., Watts J.A., Moise P.A., Finelii L., Ai C., Gupta V. (2022). A multicenter comparison of prevalence and predictors of antimicrobial resistance in hospitalized patients before and during the severe acute respiratory syndrome coronavirus 2 pandemic. Open Forum Infectious Diseases.

[186] Patel J., Sridhar D. (2022). The pandemic legacy of antimicrobial resistance in the USA. Lancet Microbe.

[187] El Omeiri N., Beith A., Bruinsma N., Caipo M., Barcos L., Mesplet M., del Barrio L., Minassian M., Arias I.C., Vásquez G. (2022). Driving multisectoral antimicrobial resistance action in South America: Lessons learned from implementing an enhanced tripartite AMR country self-assessment tool. One Health.

[188] Martin S.L., Mortimer T.D., Grad Y.H. (2023). Machine learning models for *Neisseria gonorrhoeae* antimicrobial susceptibility tests. Ann. N. Y. Acad. Sci..

[189] Coen M.E., Williford S.L., Muvva R., Genberg B., Greenbaum A., Schumacher C.M. (2023). Characteristics of reported gonorrhea diagnoses during the COVID-19 pandemic compared with pre–COVID-19 pandemic, Baltimore City, Maryland. Sex. Transmit. Dis..

[190] López-Jácome L.E., Fernández-Rodríguez D., Franco-Cendejas R., Camacho-Ortiz A., Morfin-Otero M.D.R., Rodríguez-Noriega E., Ponce-de-León A., Ortiz-Brizuela E., Rojas-Larios F., Velázquez-Acosta M.D.C. (2022). Increment antimicrobial resistance during the COVID-19 pandemic: Results from the Invifar Network. Microb. Drug Resist..

[191] Gandra S., Alvarez-Uria G., Stwalley D., Nickel K.B., Reske K.A., Kwon J.H., Dubberke E.R., Olsen M.A., Burnham J.P. (2023). Microbiology clinical culture diagnostic yields and antimicrobial resistance proportions before and during the COVID-19 pandemic in an Indian Community Hospital and two US Community Hospitals. Antibiotics.

[192] Thomas G.R., Corso A., Pasteran F., Shal J., Sosa A., Pillonetto M., de Souza Peral R.T., Hormazabal J.C., Araya P., Saavedra S.Y. (2022). Increased detection of carbapenemase-producing *Enterobacterales* bacteria in Latin America and the Caribbean during the COVID-19 pandemic. Emerg. Infect. Dis..

[193] de Souza G.H.D.A., de Oliveira A.R., dos Santos Barbosa M., Rossato L., da Silva Barbosa K., Simionatto S. (2023). Multidrug-resistant Gram-negative bacteria in patients with COVID-19: An epidemiological and clinical study. J. Infect. Public Health.

